# Systemic and Mucosal Antibody Responses to Soluble and Nanoparticle-Conjugated Antigens Administered Intranasally

**DOI:** 10.3390/antib5040020

**Published:** 2016-10-01

**Authors:** Savannah E. Howe, Gavin Sowa, Vjollca Konjufca

**Affiliations:** 1 Department of Microbiology, Southern Illinois University, Carbondale, IL 62901, USA; savannah.howe@siu.edu; 2 Department of Chemistry, Southern Illinois University, Carbondale, IL 62901, USA; gsowa49@siumed.edu

**Keywords:** antibodies, intranasal immunization, mucosal vaccines, nanoparticles

## Abstract

Nanoparticles (NPs) are increasingly being used for drug delivery, as well as antigen carriers and immunostimulants for the purpose of developing vaccines. In this work, we examined how intranasal (i.n.) priming followed by i.n. or subcutaneous (s.c.) boosting immunization affects the humoral immune response to chicken ovalbumin (Ova) and Ova conjugated to 20 nm NPs (NP-Ova). We show that i.n. priming with 20 mg of soluble Ova, a dose known to trigger oral tolerance when administered via gastric gavage, induced substantial systemic IgG1 and IgG2c, as well as mucosal antibodies. These responses were further boosted following a s.c. immunization with Ova and complete Freund’s adjuvant (Ova+CFA). In contrast, 100 µg of Ova delivered via NPs induced an IgG1-dominated systemic response, and primed the intestinal mucosa for secretion of IgA. Following a secondary s.c. or i.n. immunization with Ova+CFA or NP-Ova, systemic IgG1 titers significantly increased, and serum IgG2c and intestinal antibodies were induced in mice primed nasally with NP-Ova. Only Ova- and NP-Ova-primed mice that were s.c.-boosted exhibited substantial systemic and mucosal titers for up to 6 months after priming, whereas the antibodies of i.n.-boosted mice declined over time. Our results indicate that although the amount of Ova delivered by NPs was 1000-fold less than Ova delivered in soluble form, the antigen-specific antibody responses, both systemic and mucosal, are essentially identical by 6 months following the initial priming immunization. Additionally, both i.n.- and s.c.-boosting strategies for NP-Ova-primed mice were capable of inducing a polarized Th1/Th2 immune response, as well as intestinal antibodies; however, it is only by using a heterogeneous prime-boost strategy that long-lasting antibody responses were initiated. These results provide valuable insight for future mucosal vaccine development, as well as furthering our understanding of mucosal antibody responses.

## 1. Introduction

For many viral and bacterial pathogens that infect their hosts via mucosal surfaces of the respiratory tract there are no effective vaccines; for others, available vaccines are administered parenterally via intramuscular or subcutaneous (s.c.) injections [1]. Although this form of immunization induces substantial IgG-dominated systemic immune responses, it does not induce local secretory IgA (sIgA) antibodies, which are important for neutralizing respiratory pathogens [2,3,4,5]. For induction of local mucosal and systemic immune responses, intranasal (i.n) immunization is appealing because it is easy, and allows for increased antigen uptake via the mucosal epithelium [2,6,7,8]. Additionally, i.n. antigen administration stimulates immune responses at both local and distant mucosal sites, such as the female reproductive tract (FRT) [9,10]. The nasal mucosa and nasal associated lymphoid tissues (NALT) provide a large surface area populated abundantly by immune cells, as well as M cells, that allow for internalization of administered antigens [11,12]. However, the protective layer of mucus that covers the nasal mucosa can also trap antigens and impede their ability to reach deeper lymphoid tissues and the respiratory tract [13,14,15]. Thus poor immunogenicity of i.n.-administered antigens is often attributed to the insufficient antigen internalization. In addition, nasal administration of protein antigens was shown to induce tolerance [16,17,18], thus necessitating the coadministration of antigens with adjuvants such as cholera toxin (CT), CpG, or tetanus toxin [19,20]. Nasal coadministration of ovalbumin (Ova) and CT results in sIgA responses in various mucosal tissues as well as substantial systemic antibodies [21]; nasal administration of Ova alone results in immunological tolerance, characterized by low systemic and mucosal antibody titers and a decreased delayed type hypersensitivity (DTH) response [22,23].

For development of mucosal vaccines, nanoparticles (NPs) of various sizes and chemical compositions are being increasingly used as antigen carriers. NP properties such as size, surface charge, chemistry, material, shape, and porosity were shown to affect their immunogenicity [24]. In published reports, however, NPs larger than 200 nm have been predominantly used; they are often coadministered with adjuvants or exhibit some built-in adjuvant capacity [9,25]. Calcium phosphate NPs impregnated with influenza A hemagglutinin, when codelivered nasally with CpG, conferred protection in a mouse model of influenza virus infection [5]. N-trimethyl chitosan 300 nm NPs administered nasally were shown to induce a Th2-type dominated response, characterized by serum IgG1 and nasal IgA [26]. Despite the efficacy of adjuvants to increase the immunogenicity of nasally administered antigens, there are safety concerns associated with the use of most effective adjuvants, as they can cause damaging inflammation [27,28]. Stano and colleagues showed that Ova conjugated to 200 nm NPs given i.n. with CpG induced superior systemic and mucosal antibody responses compared to smaller NPs (30 nm), indicating that NP size is important for their immunogenicity [29]. However, smaller NPs penetrate the mucus barrier and are internalized at mucosal surfaces more efficiently than larger NPs [30,31,32]. In addition, when administered s.c., smaller NPs travel to local lymph nodes efficiently, while large NPs remain predominantly at the injection site. Similarly, the uptake of NPs by dendritic cells (DC) is significantly greater than the uptake of microparticles. In cultured cells, the optimal size for NP uptake was determined to be around 50 nm [33]. In vivo, NPs smaller than 50 nm are efficiently internalized by mucosa of the intestinal and reproductive tracts and reach the draining lymph nodes within hours of administration [34,35]. Mucosal (per-oral (p.o.) or per-vaginal) administration of Ova-conjugated 20 nm NPs (NP-Ova) without adjuvants induces a mixed systemic IgG1/IgG2c, while s.c. NP-Ova administration induces serum IgG1 antibodies only [34,35], indicating that mucosal antigen administration is important for induction of Ig isotype switching. Similarly, p.o. administration of high amounts of soluble Ova, shown to induce oral tolerance [36], induced serum IgG titers, which could not be boosted by a second s.c. immunization [35]. Additionally, p.o. priming with a high dose of soluble Ova induced Ova-specific intestinal sIgA, which was completely abrogated after s.c. boosting [35]. In contrast, intestinal sIgA became readily detectable in fecal extracts of NP-Ova-primed mice only after a second p.o.- or s.c.-boosting immunization [34,35]. Most importantly, systemic IgG1, IgG2c, and intestinal sIgA titers increased over a 6 month period in mice primed p.o. and boosted s.c., but not in mice primed and boosted p.o. [35]. These results indicate that, in addition to other factors, prime-boost immunization strategy is critical for induction of long-lasting humoral immune response. Many mucosal vaccines fail to induce immune memory, which is one of the most desirable attributes of effective vaccines. This is exemplified by rapidly waning immunity induced by the oral polio vaccine (OPV) [37].

In this work, we investigated the immunogenicity of Ova and 20 nm NP-Ova delivered nasally without the aid of mucosal adjuvants. We also report on how heterologous and homologous prime-boost strategies impact the humoral immune responses and humoral immune memory. This work will provide insight for the development of mucosal vaccines. 

## 2. Materials and Methods

### 2.1. Ethics Statement

This study was conducted in accordance with recommendations in the Guide for the Care and Use of Laboratory Animals of the National Institutes of Health. Approval of the protocol was obtained through the Southern Illinois University Institutional Animal Care and Use Committee (Protocol Number: 13-057). Animals were housed in centralized AAALAC-accredited research animal facilities, and staffed with trained husbandry, technical, and veterinary personnel.

### 2.2. Animals and Reagents

For these studies six- to eight-week-old female C57BL/6 mice (Jackson Laboratories) were used. Mice were immunized with 20 nm polystyrene NPs (Invitrogen) conjugated to Ova (Sigma, St. Louis, MO, USA) as described previously [35,38]. The amount of Ova conjugated to NPs was determined using a BCA protein assay (Pierce, Rockford, IL, USA). Mouse-specific IgG1, IgG2c, and IgA antibodies, conjugated to alkaline phosphatase (AP) (Southern Biotechnology Associates, Inc., Birmingham, AL, USA) were used to determine antibody titers in sera and mucosal secretions of immunized mice.

### 2.3. Analysis of Antigen Internalization by Immunofluorescence Microscopy (IFM)

To confirm the presence of i.n.-applied antigens within the respiratory tract, 20 nm NPs (10% from an original concentration of 2%) and soluble antigens (Ova (5 mg/20 µL, Sigma), lysine-fixable biotinylated dextran (10 K MW, Invitrogen)) were i.n. administered to anesthetized mice in a volume of 20 μL (10 μL/ nostril). At 1 h after antigen administration mice were euthanized, lung tissues were excised and snap-frozen in optimum cutting temperature (O.C.T.) freezing medium. Tissue cryosections were fixed in 10% PFA then stained with streptavidin-FITC (fluorescein isothiocyanate) and antibodies specific for Ova and DC marker CD11c (eBioscience). Tissue architecture was highlighted with actin-binding phalloidin-Alexa350 (Invitrogen). Stained tissue sections were imaged with a Leica DM1000B fluorescence microscope and acquired images were analyzed as described previously [34,38,39]. To get a more detailed view of the tissue, lung sections were also stained with hematoxylin and eosin (H&E) and imaged with an Olympus BX41 microscope equipped with an Olympus DP72 camera using CellSens Standard imaging software.

### 2.4. I.n. Immunization with Soluble Ova and NP-Ova

Mice were lightly anesthetized with isoflurane delivered in a stream of oxygen, then nasally immunized with either soluble Ova or NP-Ova (0.4% NPs in phosphate-buffered saline (PBS) from an original 2% stock solution). Antigens were delivered in a volume of 20 µL (10 µL per nostril). NP-Ova-immunized mice received doses on days 0, 1, and 2, while Ova-immunized animals received a dose on days 0, 1, 4, and 7. The total amount of Ova administered via NPs was 100 µg and the amount of soluble Ova administered was 20 mg. Four weeks after the first i.n. immunization, a group of mice primed with NP-Ova were s.c. injected with 300 µg Ova + complete Freund’s adjuvant (CFA) (Sigma) in a volume of 200 µL. Another group of NP-Ova-primed mice were i.n. boosted with one dose of NP-Ova in 20 µL (10 µL per nostril). Mice that were immunized with soluble Ova were boosted s.c. with 300 µg Ova+CFA.

### 2.5. Sample Collection and Determination of Ova-Specific Antibody Titers in Sera and Mucosal Secretions Using ELISA

Prior to i.n. priming and weekly thereafter for up to 6 months, blood, fecal pellets, and vaginal washes were collected from individual mice. Blood was collected via the lateral tail vein, while vaginal washes were collected by rinsing with 20 µL of PBS (repeated over 3 days). Fecal pellets were manually homogenized in PBS (100 mg dry matter/ml of PBS) containing 0.02% sodium azide (Sigma, St. Louis, MO, USA) and centrifuged at 10,000× *g* for 10 min in order to separate supernatant from fecal debris. Analysis of antibody titers in collected samples was done using ELISA as described previously [34,35].

### 2.6. Statistical Analysis

Each immunization experiment was repeated twice. Collected data were analyzed using ANOVA procedures of SAS software. Group means were separated using Tukey’s multiple comparison procedure and were declared significantly different at *p* < 0.05. Data are expressed as the mean ± standard deviation of the mean.

## 3. Results

### 3.1. Nasally-Applied 20 nm NPs, Ova, and Dextran Reach the Respiratory Lymph Nodes (LNs) and Lung Tissue of Anesthetized Mice

**Figure 1:**
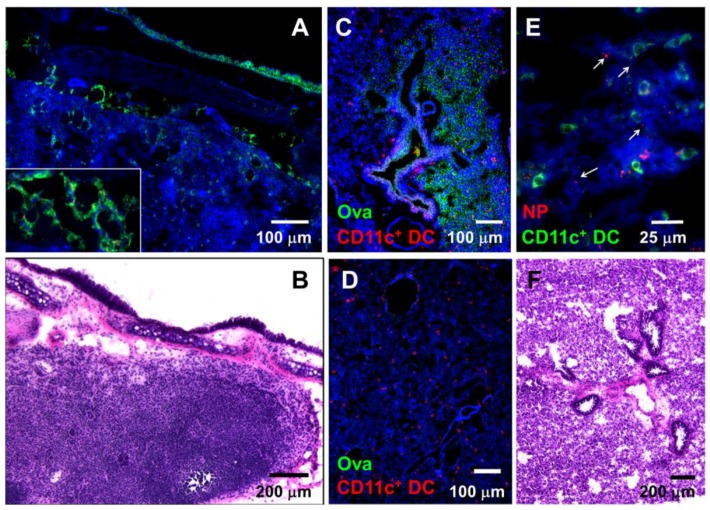
Nasally applied soluble antigens and nanoparticles (NPs) reach the mediastinal lymph nodes (LNs) and lung tissue in anesthetized mice shortly after administration. (**A**) Dextran (green) and 20 nm NPs (red) in lymph ducts of the mediastinal LNs 1 h after i.n. administration. Inset: larger magnification image showing 20 nm NPs colocalizing with dextran in lymph ducts of the mediastinal LNs. (**B**) Hematoxylin and eosin (H&E)-stained mediastinal lymph node. (**C**) Ova (green) within the lung tissue 1 h after i.n. Ova administration; CD11c^+^ DCs are shown in red. (**D**) Control lung tissue stained with antibodies against Ova and CD11c DC; (**E**) Large magnification image of lung tissue section showing 20 nm NPs (red, arrows) and CD11c^+^ DCs (green). (**F**) H&E-stained lung tissue section depicting lung tissue anatomy. (**A**, **C**, **D**, **E**) Tissue architecture was highlighted with actin-binding phalloidin-Alexa 350 (blue).

Ciliated epithelial cells of the upper respiratory tract play an important role in clearing the mucus and expelling mucus-bound particulates [40]. Similarly, mucus clearance prevents pathogens from reaching the lower respiratory tract and causing infections. Volatile anesthetics, such as isoflurane used in these studies, have been previously shown to inhibit ciliary beat frequency [41] and mucociliary clearance [42,43]. To examine whether i.n.-administered NPs and soluble antigens reach the respiratory tract, we i.n.-administered combinations of 20 nm NPs and dextran, 20 nm NPs and soluble Ova, or soluble Ova alone. Lysine-fixable biotinylated dextran was used as a soluble antigen in few experiments since it can be stained and visualized within tissues in situ with streptavidin-conjugated fluorescent probes. At 1 h after i.n. antigen administration, both NPs and soluble antigens (dextran) were found in the lymph ducts that drain into the LNs of the respiratory tract (Figure 1A,B). While dextran can be readily visualized within the lymphatic ducts that drain into the LNs, visualization of 20 nm NPs is more challenging due to their ultra-small size. Under high magnification (63X) NPs can be observed in the lymph ducts draining into the LNs, colocalizing with dextran (Figure 1A (inset),B). In the lungs, NPs were observed within the tissue (Figure 1E). Ova, administered at a concentration of 5 mg/20 µL, was also observed in the lung tissue sections (Figure 1C). Although it is difficult to quantify the total amount of Ova and NPs that reached the lower respiratory tract, it is important to point out that Ova and NPs were observed in very small areas of lung tissue sections (estimated at about 10%), indicating that a very low amount of antigen administered i.n. reaches the deeper respiratory tract. No Ova was observed in lung tissue sections of control mice (Figure 1D). The presence of Ova and NPs in the lung tissue shortly after administration is likely due to the inhibitory effect of isoflurane in mucus clearance. The nasal-associated lymphoid tissue is likely the main site where i.n.-administered antigens are internalized and warrants further investigation.

### 3.2. Nasal Administration of Soluble Ova Induces Serum IgG1/IgG2c Antibodies, While Administration of NP-Ova Prompts an IgG1-Dominated Response Prior to Boost

**Figure 2:**
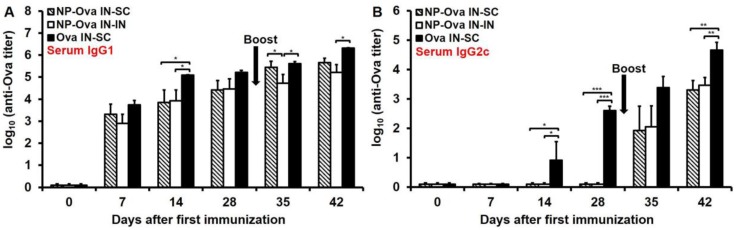
Antibody titers in sera of mice primed i.n. with soluble Ova or NP-Ova and boosted s.c. or i.n. with Ova+complete Freund’s adjuvant (CFA) and NP-Ova, respectively. Two groups of mice were i.n. immunized with 20 nm NP-Ova at day 0, 1, and 2, then boosted i.n. with NP-Ova or s.c. with Ova+CFA at day 28 (arrow). Another group of mice were i.n. immunized with 20 mg soluble Ova on days 0, 1, 4, and 7 and s.c. boosted with Ova+CFA at day 28 (arrow). Ova-specific serum IgG1 (**A**) and IgG2c (**B**) antibody titers are expressed as log_10_ titer values, with the titer being the highest dilution showing an absorbance value twice that of the negative controls. Data collected from 10 mice per group (2 separate experiments) are expressed as the mean ± SD of the mean and were declared significantly different at *p* < 0.05 (*), *p* < 0.01 (**) and *p* < 0.001 (***).

P.o. administration of high dose of Ova induces oral tolerance [22,36] characterized with suppressed cell-mediated responses. In regard to humoral immune responses, high dose of Ova (100 mg) induces serum IgG titers, which are not boosted by a second s.c. immunization. In addition, secretion of Ova-specific IgA in the intestines that is evident within a week of priming is inhibited following a s.c. antigen administration [35]. Doses of Ova ranging from 60–300 µg administered i.n. induce tolerance, characterized by very low antibody titers [44] and suppressed DTH responses [22]. Here, a high dose of soluble Ova was used in order to examine whether an antibody response could be elicited via i.n. administration as shown for p.o. Ova administration [35]. Serum IgG1 titers were measurable within one week of i.n. administration of NP-Ova (Figure 2A). Similar to a previous report [35], s.c. boosting with Ova+CFA significantly elevated serum IgG1 titers in mice i.n.-primed with NP-Ova (*p* < 0.003) (Figure 2A), but i.n. boosting did not (*p* < 0.13). However, in both groups IgG2c titers were significantly elevated after s.c. or i.n. boosting (Figure 2B, *p* < 0.01). I.n. administration of 20 mg Ova induced serum IgG1 and IgG2c titers within 7 and 14 days of priming, respectively (Figure 2A,B), which were significantly boosted after s.c. immunization with Ova+CFA at day 28 (*p* < 0.01 and *p* < 0.008, respectively).

### 3.3. I.n. Priming with High Dose of Ova, Unlike NP-Ova, Induces Intestinal IgA Which Is Not Significantly Elevated Following s.c. Boosting with Ova+CFA

**Figure 3:**
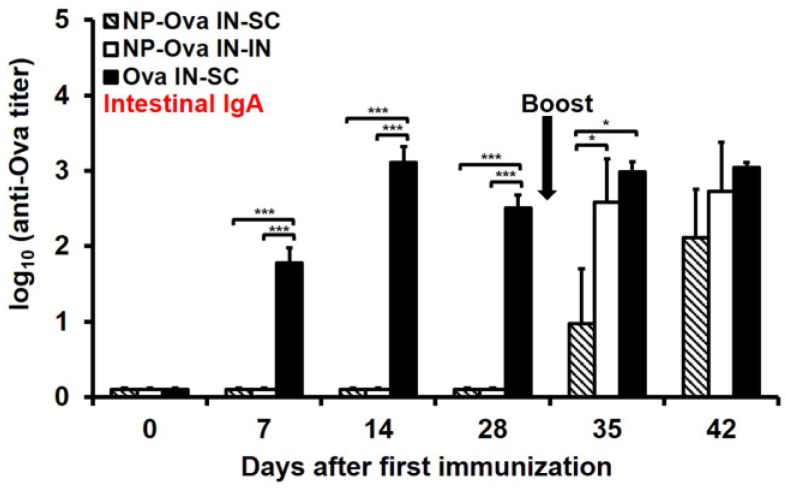
Intestinal IgA titers in mice primed i.n. with soluble Ova or NP-Ova and boosted via intranasal (i.n.) or subcutaneous (s.c.) administration with NP-Ova or Ova+CFA, respectively. Mice were i.n. immunized with NP-Ova and boosted i.n. with NP-Ova or s.c. with Ova+CFA at day 28 (arrow). Another group of mice were i.n. immunized with 20 mg soluble Ova and s.c. boosted with Ova+CFA at day 28 (arrow). Ova-specific secretory IgA (sIgA) antibody titers of fecal extracts are expressed as log_10_ titer values, with the titer being the highest dilution showing an absorbance value twice that of the negative control. Data collected from 10 mice per group (2 separate experiments) are expressed as the mean ± SD of the mean and were declared significantly different at *p* < 0.05 (*), *p* < 0.01 (**) and *p* < 0.001 (***).

Similar to p.o. priming with NP-Ova [35], i.n. priming alone was insufficient to induce an appreciable amount of IgA in fecal extracts; however, intestinal IgA titers were significantly elevated 2 weeks after either i.n. boosting with NP-Ova or s.c. boosting with Ova+CFA (Figure 3, *p* < 0.01). A large dose of soluble Ova administered i.n. induced high intestinal IgA titers within 1 week of priming similar to Ova administered per-orally [35]. IgA titers were significantly elevated following s.c. boosting (day 28 vs. day 42 titers, *p* < 0.05). This finding is in contrast to a previous report that titers of IgA in fecal extracts induced by per-oral feeding are completely abolished following s.c. administration of Ova+CFA [35].

### 3.4. Nasal Priming with Ova Induces IgA and IgG1 in Vaginal Secretions, While i.n. Priming Followed by s.c. Boosting with NP-Ova Induces IgG1 and no IgA

**Figure 4:**
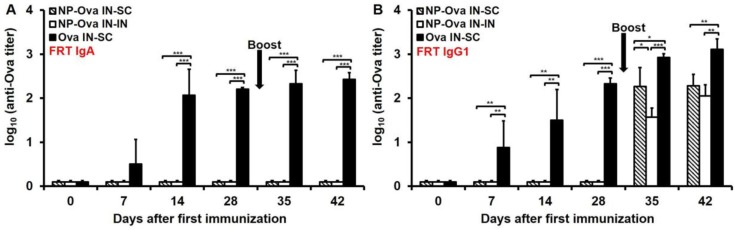
IgA and IgG1 titers in vaginal washes of mice primed i.n. with soluble Ova or NP-Ova and boosted s.c. or i.n. with Ova+CFA or NP-Ova, respectively. Mice were i.n. primed with NP-Ova then i.n. boosted with NP-Ova or s.c. boosted with Ova+CFA at day 28 (arrow). Another group of mice were i.n. immunized with Ova and s.c. boosted with Ova+CFA at day 28 (arrow). Ova-specific IgA (**A**) and IgG1 (**B**) antibody titers of vaginal washes are expressed as log_10_ titer values, with the titer being the highest dilution showing an absorbance value twice that of the negative control. Data collected from 10 mice per group (2 separate experiments) are expressed as the mean ± SD of the mean and were declared significantly different at *p* < 0.05 (*), *p* < 0.01 (**) and *p* < 0.001 (***).

Although others have shown that i.n. immunization with NPs can induce IgA in vaginal secretions [10,26,45], we could not detect sIgA in vaginal washes of mice i.n.-primed with NP-Ova prior to or after boosting (Figure 4A). In contrast, mice i.n.-primed with soluble Ova exhibited relatively high IgA titers in vaginal washes by day 14 after priming (Figure 4A), which were not significantly elevated following s.c. boosting with Ova+CFA (*p* < 0.842) (Figure 4A). In vaginal washes substantial IgG1 titers were observed within 1 week of priming with soluble Ova (Figure 4B). In vaginal washes of mice primed with NP-Ova, IgG1 was detected only after i.n. or s.c. boosting (Figure 4B), and by day 42 IgG1 titers of i.n.- and s.c.-boosted mice were not significantly different (Figure 4B), reflecting the similarities of serum IgG1 dynamics (Figure 2A).

### 3.5. Systemic (s.c.) Boosting Immunization Following i.n. Priming with NP-Ova Induces Superior Humoral Immune Memory Compared to Mucosal (i.n.) Boosting

The longevity of serum and mucosal antibody titers against Ova was evaluated for up to 180 days (6 months) after priming in order to ascertain which immunization strategy induced superior immune memory. At day 180 after i.n. priming, serum IgG1 titers of s.c.-boosted mice remained stable, while IgG2c titers significantly increased compared to their respective day-42 titers (Table 1). In contrast to this, serum IgG1 titers of i.n.-boosted mice exhibited a declining trend, while IgG2c titers decreased significantly to a point that they could no longer be detected by ELISA in all but 3 of 10 mice (Table 1). Mice primed i.n. with soluble Ova had a significant decrease in serum IgG1 titers from day 42 to day 180 after priming, but no significant change in IgG2c titers (Table 1). In all immunization groups there was no significant change in titers of IgA in fecal extracts between day 42 and 180, indicating that all prime-boost immunization strategies tested could induce long-lasting intestinal IgA, independent of their ability to ensure the longevity of systemic IgG1 and IgG2c. In the FRT secretions, IgG1 remained stable in all immunization groups, albeit in the NP-Ova (i.n.-s.c.) immunized group the IgG1 titer increase was substantial, although not statistically significant (*p* < 0.07). While no FRT IgA titers were detected at any time point in groups primed with NP-Ova, the IgA titers observed at day 42 in mice primed i.n. with Ova and s.c. boosted with Ova+CFA decreased significantly by day 180 (*p* < 0.03) (Table 1), by which time IgA was detected in only 1/3 of the mice in this group.

### 3.6. Mice Primed i.n. with Ova or NP-Ova Require a Secondary s.c. Boosting Immunization for Maintaining a Long-Lasting Mixed Th1/Th2 Immune Response

**Figure 5:**
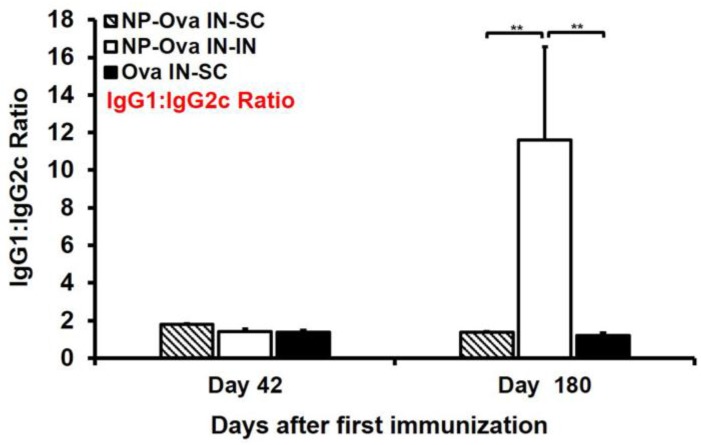
Serum IgG1:IgG2c ratio at day 42 and 180 (6 months) after i.n. priming and i.n.- or s.c.-boosting immunization. Two groups of mice were i.n. immunized with 20 nm NP-Ova at day 0, 1, and 2, then boosted i.n. with NP-Ova or s.c. with Ova+CFA at day 28. Another group was i.n. immunized with 20 mg soluble Ova on days 0, 1, 4, and 7 and s.c. boosted with Ova+CFA at day 28. The ratio of OVA-specific IgG1:IgG2c was assessed in sera of individual mice. If a particular mouse had no measureable IgG2c titers, the IgG1 value was divided by a value of 0.2 (background control number). Data collected from 10 mice per group (2 separate experiments) are expressed as the mean ± SD of the mean and were declared significantly different at *p* < 0.05 (*), *p* < 0.01 (**) and *p* < 0.001 (***).

Examination of serum IgG1 and IgG2c titers at day 28 after priming show that only mice i.n. immunized with soluble Ova exhibited a mixed Th1/Th2 response with substantial IgG1 and IgG2c titers (Figure 2A,B). Mice primed nasally with NP-Ova, on the other hand, exhibited an IgG1-dominanted serum response prior to the boosting immunization at day 28. By day 42, the IgG1:IgG2c ratios of all mice were similar (Figure 5), with no significant differences among groups, indicating that all prime-boost strategies we employed were comparable in inducing a mixed Th1/Th2 response. However, at 180 days following priming immunization, the IgG1:IgG2c ratios increased significantly only in i.n.-boosted mice due to the significant decline in serum IgG2c titers, again indicating that priming and boosting mucosally does not ensure longevity of humoral immune memory (Figure 5 and Table 1).

## 4. Discussion

Pathogen-specific antibodies induced by vaccination account for the protective efficacy of the majority of vaccines [46]. Antibodies can neutralize pathogens, mediate antigen cross-presentation, fix complement, and enhance pathogen clearance by phagocytic cells. When secreted, antibodies can prevent pathogen adhesion to mucosal surfaces, but can also enhance antigen uptake. The majority of viral and bacterial pathogens (such as influenza virus, rotavirus, norovirus, human papilloma virus, enteric bacterial pathogens, etc.) infect their hosts via mucosal surfaces, thus induction of mucosal immunity, in addition to systemic immunity, is essential for defense against such pathogens. For this, mucosal antigen administration is necessary since systemic immunization does not induce mucosal immunity. However, induction of effective mucosal immunity by vaccination is still an elusive goal for a number of reasons. First, induction of tolerance to mucosally administered antigens (p.o., i.n. and per-vaginal) must be overcome by adjuvants, the use of which is impractical for safety reasons. Second, mucosal immunity is thought to be short-lived since we have yet to figure out how to induce immune memory by mucosal vaccination, thus systemic immunization routes (s.c., intramuscular) are still the primary modes of immunization to target mucosal pathogens such as influenza and human papilloma virus. Recently we showed that mucosal (p.o.) immunization with NP-Ova without adjuvants induces a mixed serum IgG1/IgG2c, and that s.c. boosting results in superior serum and mucosal antibody titers, which remained stable for up to 6 months after the first immunization [35]. S.c. immunization alone induced IgG1-dominated serum antibodies, but no intestinal IgA, even after p.o. boosting. Using a dose and a p.o. Ova administration regimen shown to induce oral tolerance [36], we showed that soluble Ova induces secretion of intestinal IgA, which is short-lived and is completely inhibited following s.c. boosting [35]. In this work we examined whether i.n. immunization with NP-conjugated antigen is a viable approach for inducing antigen-specific mucosal and systemic antibody titers without the use of adjuvants, as reported for p.o. immunization. In addition, we examined whether systemic boosting following mucosal (i.n.) priming induces superior humoral immune memory as previously reported [35]. Since low doses of protein antigen (60–300 µg) administered i.n. induce low titers of antigen-specific serum antibodies, and tolerance to that antigen [22,23], we used a large dose of Ova (20 mg) to examine whether i.n. priming with a low Ova dose (NP-Ova) or a high dose of soluble Ova induces substantial Ova-specific antibody titers. 

We found that both Ova and NP-Ova administered i.n. to anesthetized mice are internalized at mucosal surfaces of the respiratory tract and can be found in LNs and often in lungs, colocalizing with CD11c^+^ cells. The anesthetic agent isoflurane used in these studies was shown to inhibit the mucociliary clearance, and this effect of isoflurane is likely the reason for some antigen presence in the lung tissue. In most studies for i.n. immunizations mice are anesthetized, thus the type of anesthetics used needs to be considered when comparing the results of various studies. Nasal priming with either Ova or NP-Ova resulted in significantly elevated serum IgG1 titers. In NP-Ova-primed mice, serum IgG1 was the only antibody isotype detected before boosting, while Ova-primed mice developed both IgG1 and IgG2c titers. This finding is in contrast to the findings of others that i.n. immunization with antigen alone (similar dose) induced a weak serum IgG1 and no IgG2a, while antigen coadministration with adjuvants induced mixed IgG1/IgG2a titers, demonstrating that adjuvants can influence the Th1/Th2 skewing [47]. It is well established that signaling via innate immune receptors is necessary for the development of a primary Th1 response and IgG2c antibodies to thymus-independent antigens [48]. In NP-Ova-primed mice, the boosting immunization (s.c. or i.n.) caused isotype switching, resulting in not only high serum IgG2c but also intestinal IgA. Our findings argue that while antigen presentation in the presence of innate receptor stimulation provided by microflora is sufficient to induce isotype switching, additional factors such as antigen dose and priming site also play a role. Perhaps when antigens are targeted to the nasal and bronchiolar lymphoid tissues, a higher antigen dose is required for inducing the isotype switch. In addition, mucosal surfaces of the respiratory tract are exposed to lesser or no microbial stimulation compared to the intestinal mucosa, which can also affect isotype switching. After either i.n. or s.c. boosting, all immunization groups developed a balanced Th1/Th2 responses, exemplified by comparative IgG1/IgG2c ratios. Interestingly, by 6 months, mice primed and boosted i.n. had drastically higher IgG1/IgG2c ratios, reflecting the significant decline of serum IgG titers (especially IgG2c) in this group.

It is known that i.n. immunization can induce IgA secretion at distal mucosal sites such as the FRT and the gastrointestinal tract [10,26,45,49]. We found that only soluble Ova induced substantial IgA titers in fecal extracts, as well as IgA and IgG1 in the FRT by week 1 after i.n. priming. NP-Ova induced intestinal IgA and FRT IgG1 only after boosting, and no IgA was found in the FRT at any time point. In this work, Ova and NP-Ova were administered in a commonly used volume of 20 µL (10 µL/nostril) and although we show that both Ova and NPs can reach the respiratory tract, it is possible that a fraction of i.n. applied antigens reached the intestines where T-independent IgA class-switch recombination might have occurred as shown previously [35]. However, in mice primed i.n. with a high dose Ova, intestinal IgA was boosted after s.c. immunization, rather than abrogated when Ova was administered [35]. Used at a high concentration, it is possible that Ova aggregates and organizes as a nanoparticle, thus affecting its uptake and immunogenicity. Others have shown that lung CD103^+^ DCs can induce IgA class-switch recombination by activating B cells via T cell-dependent and T cell-independent mechanisms [49] and can imprint α4β7 and CCR9 gut-homing receptors on local IgA-expressing B cells, which then migrate to the gut where they impart protection against an enteric toxin challenge [49]. In these studies [49], a volume of 40 µL antigen solution was used for i.n. immunizations after it was established that it targets lung DCs more effectively compared to a volume of 5 µL. Using immunofluorescence microscopy, we did observe NPs and Ova in various compartments of the respiratory tract; however, we also observed some NPs and Ova in the intestines (not shown). Whether a fraction of NPs and Ova reached the intestines via an internal antigen transport route (e.g., by CD103^+^ DCs) after uptake in the nasal and bronchiolar lymphoid tissue, or whether a fraction of these antigens were simply swallowed remains to be determined.

Induction of IgA responses in the FRT by conventional immunization has been challenging, however many groups have demonstrated that this can be accomplished by i.n. immunization. For example, i.n. (as well as p.o.) immunizations with antigen-conjugated 300 nm NPs [45] resulted in IgA titers within the FRT; however, this response offered partial protection following intra-vaginal challenge with SHIV in macaques [50]. Intranasal immunization with elementary bodies (EB) of *Chlamydia trachomatis* mouse pneumonitis biovar induced IgA and IgG antibodies in the FRT and provided protection following a genital challenge 6 months after priming. However, at this time vaginal IgG and IgA titers were very low and were halved compared to titers at 2 days and 4 months after immunization, respectively [51]. Therefore, it is evident that i.n. immunizations can induce protective antibody responses in distant mucosal sites (gut, FRT); however, much remains to be learned about how the nature of antigen, antigen dose, and prime-boost immunization affect the magnitude, and especially the longevity, of these responses. By administering NP-Ova in the FRT we failed to induce IgA titers in vaginal washes that were quantifiable by ELISA, although the presence of some IgA in vaginal washes was detected by western blot [34]. Here we show that i.n. immunization with a high dose of Ova does induce high titers of FRT IgA (log_10_ of 2.43 or 1:300 dilution), however this response is short-lived as IgA titers decline significantly by 6 months after priming to a positive titer at 1:10 dilution (log_10_ of 0.83). Perhaps lung- or gut-induced IgA-secreting B cells migrate to the FRT and populate it for a finite period of time. Since we did not detect IgA titers in lung lavages of NP-Ova-immunized groups (data not shown), it could be that IgA secreted in lungs is short-lived, much like IgA in the FRT, and by 6 months becomes undetectable. We have found that p.o. prime-boost can induce IgA secretion in the FRT; however, we suspect that this response may also be short-lived (ongoing work), as reported here for i.n. immunization.

Most importantly, we show that mucosal prime-boost (i.n.–i.n.) induces inferior immune memory, reflected in serum IgG1 and IgG2c titers that decline drastically by 6 months. Similarly, in mice primed with soluble Ova, serum IgG1 and FRT IgA and IgG1 titers decline drastically by 6 months. In fact, only mice primed i.n. with NP-Ova and boosted s.c. with Ova+CFA showed no decrease of serum or mucosal antibody titers for up to 6 months after priming. Moreover, in this group, IgG1 titers in the FRT and IgG2c titers in serum increased significantly over time. Ova-specific intestinal IgA titers remained stable for the entire 6 month period in all immunization groups, as reported previously [35]. 

Immunity induced by a single or multiple p.o. (mucosal) vaccinations with OPV is incomplete and wanes rapidly [37]. Our results are in agreement with and provide insight for the success of inactivated polio vaccine (IPV) in boosting the immunity among children with a history of multiple OPV doses [52]. Immunization recommendations by the World Health Organization (WHO) for the eradication of polio includes the introduction of at least one dose of IPV into routine immunization schedule [53]. This work confirms our previous reports that mucosal priming, followed by a systemic (s.c.) boosting, is the most effective immunization strategy for inducing long-lasting mucosal and systemic humoral immunity. We believe this work will be important not only for understanding how humoral immune memory is regulated, but also for the development of much needed mucosal vaccines.

## 5. Conclusions

In conclusion, we show that (1) Ova and NP-Ova are efficiently internalized when administered nasally; (2) both homologous and heterologous boosting immunizations of NP-Ova-primed mice induce isotype switching, serum IgG2c, and intestinal sIgA; (3) heterologous boosting immunization induces a superior immune memory; and (4) high dose of soluble Ova administered i.n. induces IgA in the FRT, which is short-lived.

## Figures and Tables

**Table 1:** Systemic and mucosal Ova-specific antibody titers at day 42 and 180 after priming immunization.

Immunization Strategy	Isotype	Log_10_ Ova-specific antibody titer by ELISA ^a^		
		Day 42	Day 180	*p*
1° NP-Ova I.N 2° Ova+CFA S.C.	Serum IgG1	5.66 ± 0.4	5.71 ± 0.5	0.8
	Serum IgG2c	3.30 ± 0.7	4.30 ± 0.8	0.04
	Intestinal IgA	2.12 ± 1.3	2.15 ± 1.0	0.9
	FRT IgA	0.00 ± 0.0	0.00 ± 0.0	1.0
	FRT IgG1	2.28 ± 0.5	3.41 ± 1.3	0.07
1° NP-Ova I.N. 2° NP-Ova I.N.	Serum IgG1	5.21 ± 0.7	4.32 ± 0.9	0.08
	Serum IgG2c	3.47 ± 0.5	1.00 ± 1.6	0.001
	Intestinal IgA	2.73 ± 1.3	2.87 ± 0.9	0.9
	FRT IgA	0.00 ± 0.0	0.00 ± 0.0	1.0
	FRT IgG1	2.05 ± 0.5	1.90 ± 0.4	0.7
1° Ova I.N. 2° Ova+CFA S.C	Serum IgG1	6.31 ± 0.0	5.73 ± 0.3	0.01
	Serum IgG2c	4.67 ± 0.5	4.71 ± 0.5	0.9
	Intestinal IgA	3.04 ± 0.1	3.04 ± 0.4	1.0
	FRT IgA	2.43 ± 0.3	0.82 ± 1.2	0.03
	FRT IgG1	3.10 ± 0.5	2.87 ± 1.2	0.7

^a^ Data are expressed as the mean ± SD of the mean. Group means were separated using Student’s *t*-test.

## Abbreviations

The following abbreviations are used in this manuscript:

I.n.: intranasal
S.c.: subcutaneous
P.o.: per oral
NPs: nanoparticles
Ova: ovalbumin
NP-Ova: Ova conjugated 20 nm NPs
Ova+CFA: Ova with complete Freund’s adjuvant
NALT: nasal associated lymphoid tissue
DC: dendritic cells
LNs: mediastinal lymph nodes
FRT: female reproductive tract
CT: cholera toxin
OPV: Oral polio vaccine
IPV: Inactivated polio vaccine
DTH: Delayed Type Hypersensitivity
